# Parallel Explicit and Implicit Control of Reaching

**DOI:** 10.1371/journal.pone.0007557

**Published:** 2009-10-22

**Authors:** Pietro Mazzoni, Nancy S. Wexler

**Affiliations:** 1 Motor Performance Laboratory, Department of Neurology, Columbia University, New York, New York, United States of America; 2 Departments of Neurology and Psychiatry, Columbia University, New York, New York, United States of America; 3 Hereditary Disease Foundation, New York, New York, United States of America; Victoria University of Wellington, New Zealand

## Abstract

**Background:**

Human movement can be guided automatically (implicit control) or attentively (explicit control). Explicit control may be engaged when learning a new movement, while implicit control enables simultaneous execution of multiple actions. Explicit and implicit control can often be assigned arbitrarily: we can simultaneously drive a car and tune the radio, seamlessly allocating implicit or explicit control to either action. This flexibility suggests that sensorimotor signals, including those that encode spatially overlapping perception and behavior, can be accurately segregated to explicit and implicit control processes.

**Methodology/Principal Findings:**

We tested human subjects' ability to segregate sensorimotor signals to parallel control processes by requiring dual (explicit and implicit) control of the same reaching movement and testing for interference between these processes. Healthy control subjects were able to engage dual explicit and implicit motor control without degradation of performance compared to explicit or implicit control alone. We then asked whether segregation of explicit and implicit motor control can be selectively disrupted by studying dual-control performance in subjects with no clinically manifest neurologic deficits in the presymptomatic stage of Huntington's disease (HD). These subjects performed successfully under either explicit or implicit control alone, but were impaired in the dual-control condition.

**Conclusion/Significance:**

The human nervous system can exert dual control on a single action, and is therefore able to accurately segregate sensorimotor signals to explicit and implicit control. The impairment observed in the presymptomatic stage of HD points to a possible crucial contribution of the striatum to the segregation of sensorimotor signals to multiple control processes.

## Introduction

We can perform most everyday movements either automatically or while paying attention to how we move. We can mindlessly reach for a light switch while walking into a room and simultaneously carry out a conversation on a mobile phone, or we can guide the same movement with full attention to this action, while doing nothing else. In the first case, the action of reaching for the light switch is thought to be guided by implicit control, which is abstract and unavailable to consciousness [1], [2], [3], [4], and which is typically engaged in “automatic” movements, such as steering a car. In the second instance, reaching is thought to be under explicit control, which is rule-based and available to consciousness [1], [2], [5], and which is typically engaged by unfamiliar tasks, such as playing a piano scale for the first time. Explicit control is usually invoked when first learning a new motor skill, while implicit control characterizes movements after they have been fully learned [5], [6].

An interesting aspect of implicit control is that we are able to optionally devote attentive guidance to a movement that we can already perform automatically. This ability implies that the relationship between control processes and movement execution is not obligatory: the same movement may be guided implicitly or explicitly. Given that implicit and explicit control processes may be subserved by distinct neural networks [7], [8], [9], the implication with regard to neural substrate is that distinct neural processes can guide the same movement.

If implicit and explicit motor control represent distinct neural operations, then the nervous system must have mechanisms for routing sensorimotor signals to and from these separate control processes. The existence of such mechanisms is suggested not only by the ability to control movements explicitly or implicitly, but also by evidence that these control processes may guide movements simultaneously. We recently showed that implicit motor control cannot be disengaged voluntarily during visuomotor adaptation [10]. Therefore, when we explicitly control a movement that we could otherwise perform automatically, implicit control processes may continue to influence movement. This suggests that the motor system is able to exert dual control on one action, and can thus segregate sensorimotor signals related to one action across multiple parallel control processes.

**Figure 1:**
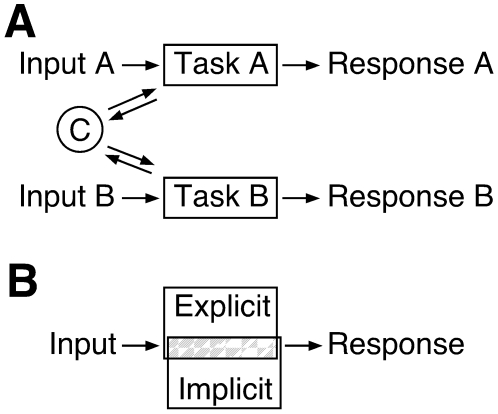
Schematic illustration of two types of dual-process experimental protocols. (A) Dual-task protocol. Each task operates on its own input (sensory or stored information) and produces its own response. C ( =  common resources) represents processing resources, such as attention, shared by the two tasks. (B) Dual-control protocol. Each control process receives the same input, and the resulting response is a single one that is influenced by both control processes. Grey area indicates shared sensorimotor information manipulated by each process.

We tested, in healthy human subjects, whether the motor system can guide a single movement through parallel explicit and implicit control processes. We devised a dual-control reaching task in which movement direction was controlled by both visuomotor adaptation and spatial working memory processes. Our task differed from typical dual tasks, in which two separate actions are executed simultaneously (for example, detecting the appearance of a letter on a screen while tapping a finger). In such tasks, input and response domains are separate, and the tasks can potentially be controlled in parallel without intermingling of control signals (Fig. 1A). In contrast, we designed a task so in which two control processes had to guide the same action, thus requiring segregation of sensorimotor signals to parallel control processes (Fig. 1B). We then looked for selective disruption of this segregation in subjects with the genetic mutation for Huntington's disease (HD) but with no clinically manifest neurologic deficits.

We chose HD, an inherited neurodegenerative disorder characterized by progressive degeneration of the striatum [11] and areas of cerebral cortex [12], on clinical and anatomical grounds. This disease causes adult-onset progressive motor, cognitive, and psychiatric symptoms [13], including abnormalities in reaching movement kinematics [14], sensorimotor error correction [15], motor skill learning [16], and simultaneous performance of multiple tasks [17], dividing attention [18], and explicit learning of motor sequences [19]. We considered the possibility that impaired segregation of sensorimotor signals constitutes a low-level deficit that could contribute to some of these impairments.

The anatomical motivation for choosing HD is that the striatum, a major site of neurodegeneration in HD, is a natural candidate structure for sensorimotor signal segregation. It receives inputs from most areas of the cerebral cortex, and influences, through the globus pallidum and thalamus, frontal areas involved in motor control. Its connectivity is characterized by segregated parallel pathways to and from cortex and by convergence, within pathways, of signals across many cortical areas [20], [21], [22]. These features make the striatum a potential critical node for segregation of sensorimotor control signals to separate control processes, as it is anatomically poised to monitor sensory, motor, and cognitive signals, and to combine and segregate these signals through specific pathways to influence processing in several cortical areas.

Because motor deficits could confound our results, we did not test patients with manifest (symptomatic) HD. We instead tested pre-symptomatic individuals with the genetic mutation for HD (asymptomatic carriers; AC), who did not have any clinical manifestations of HD. Because the HD mutation has 100% penetrance, these individuals all develop clinically evident HD at some point in their lives. We hypothesized that a deficit in sensorimotor signal segregation may be less apparent than clinical movement abnormalities, and thus may be present in pre-symptomatic individuals.

## Materials and Methods

### Ethics Statement

All study subjects gave informed consent. Testing was performed with approval by Columbia University's Internal Review Board and in accordance with the Declaration of Helsinki.

### Subjects

We tested 28 participants (Table 1) selected from the cohort of individuals enrolled in the International Venezuela Huntington's Disease Collaborative Research Project in Maracaibo, Venezuela. The Maracaibo cohort consists of approximately 14,000 individuals in families with members affected by HD, and includes patients with clinically manifest HD (not included in our study), individuals with the genetic mutation for HD who have not yet developed clinical manifestations (gene-positive carriers; asymptomatic carriers, or *AC subjects*), and mutation-negative individuals (*control subjects, CTL*) from the same community as AC subjects. Neurologic examinations were performed by neurologists with specialized training in assessing HD, who had no knowledge of subjects' participation in our study. All AC subjects in our study had a score <3 on a quantitative neurologic examination for HD (maximum possible score  = 204; higher score indicates greater severity) [23] and were thus neurologically normal, and no history of musculoskeletal disease.

**Table 1:** Study participants and Testing Protocols.

Group	Subject Type	Protocol	Conditions	Gender	Age	CAG repeat length
I	CTL	A	BL, ROT	F	32	18
I	CTL	A	BL, ROT	F	29	21
I	CTL	A	BL, ROT	M	32	20
I	CTL	A	BL, ROT	F	39	24
I	CTL	A	BL, ROT	M	26	22
I	CTL	A	BL, ROT	M	25	19
*n = 6*				*mean±SD:*	*31±5*	
II	AC	A	BL, ROT	F	34	46
II	AC	A	BL, ROT	F	37	42
II	AC	A	BL, ROT	F	24	40
II	AC	A	BL, ROT	F	24	43
II	AC	A	BL, ROT	F	27	46
II	AC	A	BL, ROT	F	32	47
II	AC	A	BL, ROT	M	44	42
*n = 7*				*mean±SD:*	*32±7*	
III	CTL	B	OB, OB+ROT	F	33	17
III	CTL	B	OB, OB+ROT	F	24	21
III	CTL	B	OB, OB+ROT	F	46	23
III	CTL	B	OB, OB+ROT	M	23	21
III	CTL	B	OB, OB+ROT	F	24	24
III	CTL	B	OB, OB+ROT	F	22	21
III	CTL	B	OB, OB+ROT	F	27	19
*n = 7*				*mean±SD*	*28±9*	
IV	AC	B	OB, OB+ROT	M	29	46
IV	AC	B	OB, OB+ROT	F	30	39
IV	AC	B	OB, OB+ROT	F	28	44
IV	AC	B	OB, OB+ROT	F	31	42
IV	AC	B	OB, OB+ROT	M	19	52
IV	AC	B	OB, OB+ROT	F	24	39
IV	AC	B	OB, OB+ROT	F	24	42
IV	AC	B	OB, OB+ROT	M	27	48
*n = 8*				*mean±SD:*	*27±4*	

*CAG repeat length*, number of CAG trinucleotide repeats in mutated allele;

*CTL*, control; *AC*, asymptomatic carriers; *BL*, baseline; *ROT*, rotation; *OB*, one-back.

Subjects were divided into four groups. Groups I (CTL) and II (AC) were tested in Protocol A (see below), while groups III (CTL) and IV (AC) were tested in Protocol B (Table 1). There was no significant difference in age between groups whose performance was compared: groups I and II (*p* = 0.73; 2-sample *t* test), III and IV (*p* = 0.60), and II and IV (*p* = 0.13). One researcher (author PM) carried out all data collection and kinematic data processing without knowledge of subjects' gene status.

### Apparatus

**Figure 2:**
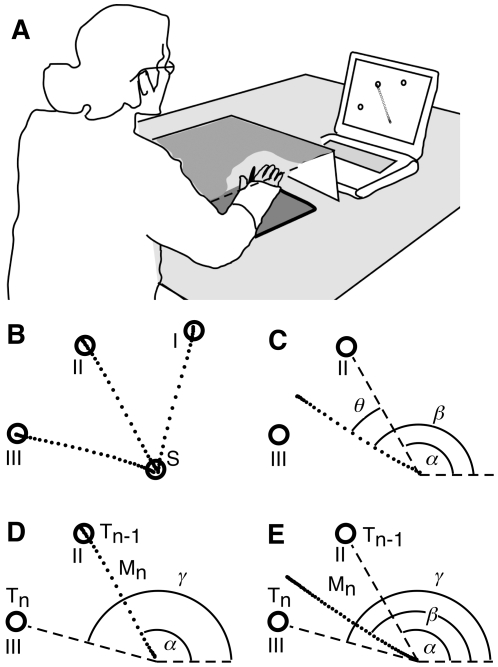
Apparatus and motor tasks. (A) Subject sitting at a table performing the baseline task. The right hand is in a wrist splint with an attached stylus, whose position is recorded by a graphics tablet. Vision of the hand is blocked by a cardboard box. A laptop computer shows the visual targets as circles and the hand's position as a cursor. (B) Sample cursor paths (dots) for three trials (one to each target direction) in the BL condition. Targets I, II, and III are arranged at 75°, 120°, and 165°, respectively, relative to the starting circle (S). (C) Sample cursor path for a trial early in the ROT condition. Target II appears (direction *α* ) and the subject's hand moves in this direction. Due to the imposed 30° CCW rotation, however, the cursor moves along direction *β*. The angle between the cursor's path and the target's direction is the directional error (*θ = β − α*). Between trials, this error induces adjustments of the visuomotor map that are reflected in the next movement. (D) Sample path in the OB condition. The subject is remembering that the previously shown target was II (T_n−1_; direction *α* ). The current target (T_n_) appears at 165° (direction *γ* ). The subject makes a movement in direction *α* . Between trials, the subject holds in memory direction *γ* for the next movement. (E) Sample cursor path for an early trial in the OB+ROT condition. The subject is remembering that the previously shown target was target II (T_n−1_; direction *α* ). The current target (T_n_) appears at 165° (direction *γ* ). The subject makes a movement in direction *α* , and memorizes direction *γ* for the next movement. However, due to the imposed 30° CCW rotation, the cursor's path is in direction *β*. Between trials, therefore, the subject must also process the directional error (*θ = β − α*), while holding in memory the target's direction (*γ*).

Subjects sat at a table facing a laptop computer (Fig. 2) and moved their dominant arm on a digitizing tablet (Wacom ArtZ II graphics tablet, 9×12 inches, Saitama, Japan). They could not see their hand, which was splinted to prevent wrist motion. The tablet recorded hand position through a stylus attached to the splint. Two Teflon-coated discs attached to the splint allowed comfortable sliding motion of the hand over the tablet with little friction. A laptop computer (Powerbook G3, Apple, Cupertino, CA) displayed visual stimuli and recorded hand position data at 50 Hz (Fig. 2A). The display included a circular cursor indicating current hand position, a starting circle (2-cm diameter), and three target circles (2-cm diameter; Fig. 2B). The display's scale was matched to the tablet so that movement amplitudes were the same for cursor and hand.

In all conditions, the basic task was to move the hand on the tablet so as to guide the cursor shown on the display from the starting circle to a visual target. To start each trial, subjects moved their hand so as to place the cursor inside the starting circle shown on the computer display. One second later a circular target appeared, accompanied by a tone, at a distance of 8 cm from the start circle, in one of three possible directions (Fig. 2B): 75°, 120°, and 165° (where 0° is the “3 o'clock” direction). Subjects were instructed to make fast straight out-and-back hand movements toward the target (selected according to each condition) without changing direction during the movement. If the cursor hit the target, the target changed color (from red to green) and a gunshot-like sound was played. Movements were made in blocks of trials in which targets appeared in pseudorandom sequence without trial-to-trial repetition.

Note that the instruction was to start each movement only when ready, and there was no penalty for starting a movement later. Thus, although subjects made fast movements, they had ample time for movement preparation.

### Conditions

#### Baseline (BL)

Subjects were instructed to place the cursor in the starting circle and wait for one of the targets to appear, at which point they were to make a single arm movement rapidly (but not as fast as possible) when ready (not as soon as possible), so as to bring the cursor into the target that just appeared, and to immediately return to the start circle without stopping in the target (“out-and-back” movement). Sample cursor paths for this condition are shown in Fig. 2B.

#### Rotation (ROT; implicit control)

The cursor was displayed at a position that was rotated, relative to the start circle, from the hand's actual position by a 30° angle in the counterclockwise (CCW) direction. Subjects were warned in advance that “the computer might do something strange”, and that they should keep making the same type of movements (fast, out-and-back, without corrections during the movement) while still aiming to hit the target. Fig. 2C shows a sample cursor path from a trial in the early phase of the ROT condition. The hand moves towards target II (hand path not shown); the cursor's path is rotated by angle *θ* relative to the hand path.

#### One-back reaching (OB; explicit control)

The instruction was to move the cursor into the target that had just appeared on the previous trial (Fig. 2D). (For the first trial the instruction was not to move but simply observe where the target appeared). This condition was modeled on the “*n*-back” task employed to study working memory [24], and we used it as a spatial working memory task. It requires choosing movement direction by following an explicit rule under conscious awareness. It is closely related to the one-back choice reaction time task [25], which was shown to engage working memory.

#### One-back + rotation (OB+ROT; dual control)

In this condition a 30° CCW rotation was imposed while subjects made reaching movements to the previous target, according to the one-back requirement. This condition required simultaneous engagement of explicit and implicit control of movement direction (Fig. 2E).

The above conditions manipulated the control mode for selecting movement direction. In each condition other than BL, the task required moving the hand in a direction different from that of the target. In ROT, target direction had to be mapped to a rotated direction of hand movement. This adaptation entailed comparing, between movements and under implicit control, the planned movement direction with the resulting cursor's direction, and to incrementally modify an internal model of visuomotor space [26]. We recently demonstrated that adaptation to a visuomotor rotation occurs under implicit control: this type of learning proceeds at normal rates even when subjects try, through a conscious strategy, to prevent adaptation from occurring [10]. Additional evidence that this type of learning is implicit is the gradual decay (after-effect) of rotation learning after the rotation is removed [27].

In OB, subjects had to plan a different movement direction relative to the target's direction. The one-back component required, between each movement, maintaining in working memory the direction of the previous target; planning the next movement based on this previous direction; and replacing it with the next target's direction when it appeared. This type of working memory task is widely considered to be guided by conscious awareness [28], [29] and thus is under explicit control.

In OB+ROT, there was full overlap of stimulus and response domains: the same visuospatial information (target direction) was used by each control process, and both processes controlled a single response variable (movement direction). The OB+ROT condition thus involved dual control of a single sensorimotor behavior by parallel explicit and implicit control processes.

### Testing Protocols

Because rotation learning occurs faster when a subject performs it for a second time, we did not test the same subjects in the ROT and the OB+ROT conditions, so as to avoid the confounding effect of repeated exposure to rotation learning. We thus divided testing conditions into two protocols (A and B), and divided each subject group (controls and AC) into two groups (Table 1). All subjects first performed 6–9 movements in the BL condition to familiarize themselves with the apparatus and the basic motor task.

#### Protocol A

After familiarization with the apparatus, groups I (CTL; N = 6) and II (AC; N = 7) performed 24 trials in condition BL, immediately followed by ROT (69 trials) and then BL (39 trials).

#### Protocol B

After familiarization with the baseline task, groups III (CTL; N = 7) and IV (AC; N = 8) were familiarized with the OB condition (6–9 trials), and were then tested in the OB condition (24 trials) immediately followed by OB+ROT (60 trials).

### Data Analysis

Kinematic data was analyzed offline using custom software written in IGOR software (Wavemetrics, Lake Oswego, OR). Position data was first filtered using a smoothing spline algorithm with factor 0.1 and then differentiated to obtain tangential velocity. *Movement start time* was determined by first identifying *peak velocity* (first maximum above 15 cm/s) and then searching backward along the velocity trace for the first value below a threshold of 1 cm/s. *Movement end time* was identified as the first minimum to the right of the occurrence of peak velocity. We defined a workspace with origin at the center of the start circle, and *x*–*y* axes parallel to the tablet's borders. Movement start and end positions were defined as the hand's position at the time of movement start and end, respectively. *Cursor direction* was the angle between the *x* axis and the line connecting the origin and each movement's end position (*β* in Fig. 2C). *Target direction* was the angle between the *x* axis and a line connecting the origin and the target's center (*α* in Fig. 2C). *Directional error* (*θ* ) was defined as the difference between cursor direction and target direction (*β* – *α* in Fig. 2C). We also considered a measure of directional error based on cursor direction at peak velocity, rather than at movement endpoint. However, this measure produced equivalent results (as expected, given that movements were largely straight), and is thus not reported with our results. Other kinematic measures are described in Supporting Text S1.

Statistical tests included *t* tests, ANOVA, and linear regressions, performed with JMP software (SAS Institute, Cary, NC). These are listed for specific analyses in *Results*.

### Identification of Implicit and Explicit Control Errors

**Figure 3:**
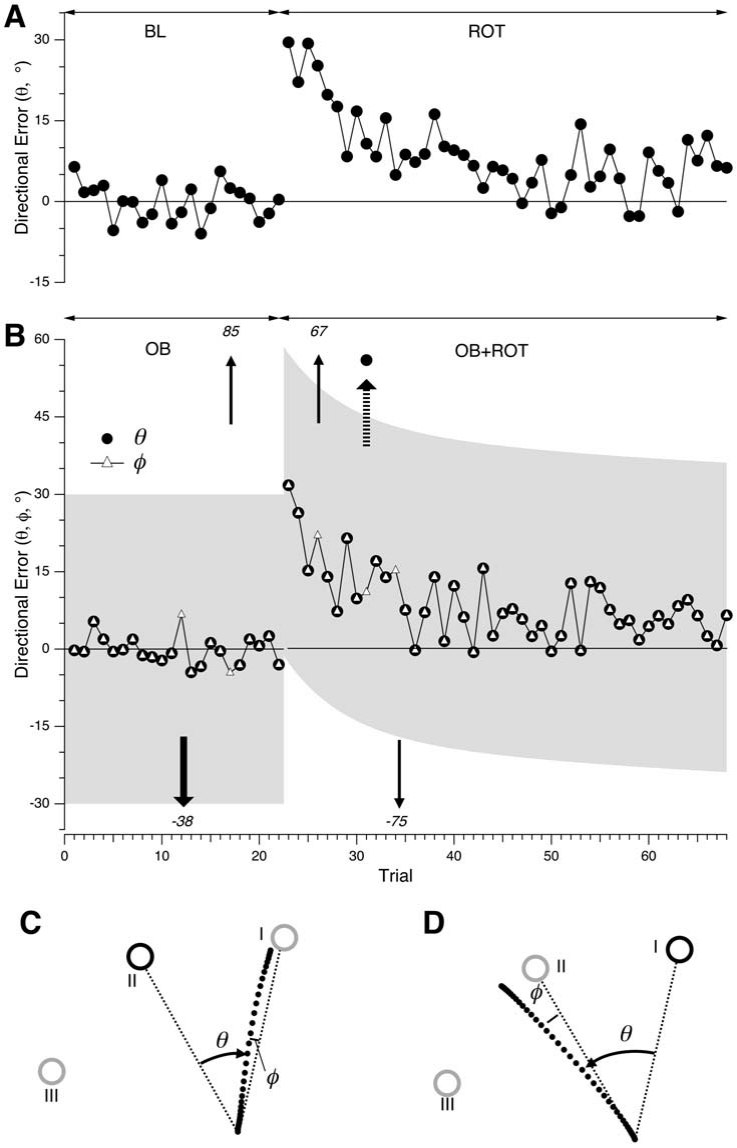
Directional error of two representative CTL subjects in a series of trials in each task condition. (A) Directional error vs. trial number in the baseline (BL) and rotation (ROT) conditions for a CTL subject in group I. Error *θ* is the angle, in degrees, between target and cursor directions (see Fig. 2C). A 30° rotation was imposed at trial 23. (B) Directional error (*θ*; filled circles) and adjusted directional error (*φ*; open triangles) vs. trial number in the one-back (OB) and dual task (OB+ROT) conditions for a group III CTL subject. The trials shown are a portion of a testing session. Arrows indicate instances where *θ* differed from *φ*; such values of *θ* are indicated in text near the arrows, because they fall outside the range shown in this plot. Grey shaded regions indicate range of plausible directional errors (see text). (C) Cursor path for trial 12 (wide downward arrow in Fig. 3B), illustrating the calculation of adjusted directional error (*φ* ) in an OB trial. The current target is I (grey open circle), while the previous target is II (black open circle). Because of the OB condition, the correct target for the movement is II. While *θ* is large (outside the range of plausible errors), *φ* is small, indicating that the subject likely selected target I (target selection error). (D) Cursor path for trial 31 (wide dashed upward arrow in Fig. 3B), illustrating the calculation of adjusted directional error (*φ*) in an OB+ROT condition. The current target is II (grey open circle), while the previous target is I (black open circle). The correct target for the movement is I. *θ* is large (greater than the separation between targets), while *φ* is within the range expected during rotation learning. This indicates that the subject likely (incorrectly) selected target II.

Cursor direction was influenced both by implicit control (adaptation to visuomotor rotation) and explicit control processes (target selection based on the one-back rule). We devised the following procedure to disambiguate the nature (implicit vs. explicit) of directional error. Fig. 3 shows single-subject examples of directional error for several movements in each of the four conditions used in our study. In the BL condition, the subject moves the cursor to the current target, and errors clustered around 0° with some variability across trials (Fig. 3A, first 22 trials). At trial 23 a 30° rotation was imposed (ROT condition), and directional error (*rotation error*) initially jumped to around 30°. With repeated trials, the subject learned the new mapping, and directional error gradually decreased. In the last 10 trials of the segment shown the error decreased to the range 5–10°.

Directional errors for a different subject are shown in Fig. 3B. The first 22 trials are in the one-back condition (OB): the instruction is to make movements to the target shown in the previous trial. This instruction required the subject to maintain in working memory the previous target's direction. Black circles show the directional error *θ*, which is the angle between the current cursor path (M_n_ in Fig. 2D) and the previous target's direction (T_n−1_ in Fig. 2D). Similarly to the BL condition, the error varied about a mean position of 0 over a range of approximately ±7°.This indicates successful performance of the one-back task. Errors in following the one-back instruction resulted in wrong target selection, such as selecting the current target, T_n_, instead of the previous target, T_n−1_ (Fig. 2D). We reasoned that errors with magnitude far outside the range expected on baseline performance must reflect errors in target selection, or *target errors*.

Target errors are also demonstrated in Fig. 3B (arrows). On these trials, *θ* assumed values far outside the baseline range. Such large errors are consistent with selection of the wrong target. Fig. 3C depicts the cursor path for trial 12 of Fig. 3B. Whereas the appropriate target (the target shown on the previous trial) was II (Fig. 3C), the cursor's direction was effectively along the direction of target I (the current target). The subject thus failed to follow the one-back rule: she made a movement to the current target rather than to the previous one. Directional error relative to target I is small (9.3°), well within trial-to-trial variability. We took this as evidence that the subject likely aimed her movement at target I instead of II. Similarly, the error on trial 17 could be reduced from 85° to −5° by replacing the actual target with target III. (Note that the apparently chosen target was not always the current target.)

Target errors in the OB condition were thus directional errors that far exceeded baseline variability, computed as standard deviation of directional error in BL (5°±2°, mean±SE across all groups; no significant difference between CTL and AC groups). We defined a range of directional errors (*plausible range*) outside of which any error was considered a target error. We set the width of this range at ±6× the baseline standard deviation (±30°; shaded band in Fig. 3B) in order to minimize the chance of incorrectly assigning a target error.

**Figure 4:**
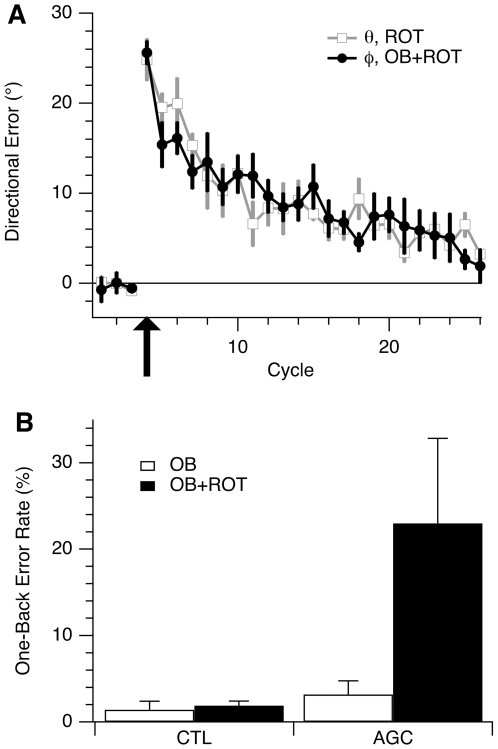
Performance in single- and dual-control tasks. (A) Learning curves of CTL subjects exposed to visuomotor rotation in single- and dual-control conditions. Open squares (grey trace) show directional error (*θ*, as defined in Fig. 2C), averaged within each cycle (1 cycle = 3 consecutive trials) and across all subjects in group I, vs. cycle number in the ROT condition. Filled circles (black trace) show the adjusted directional error (*φ*, as defined in Fig. 3D), averaged within each cycle and across all subjects in group III, vs. cycle number in the OB+ROT condition. The first three cycles were performed without rotation. At cycle 4 (arrow) a 30° CCW rotation was imposed for the remainder of the session. Error bars indicate standard error for the subject group. (B) Percentage of target selection errors for CTL and AC subjects in the single-control version of the one-back condition (OB) and the dual-control version (OB+ROT). Bars indicate group mean±s.e.

Trials 23–71 in Fig. 3B show the subject's performance when both rotation and one-back conditions are combined (condition OB+ROT). The directional error *θ* jumps to a value near 30°, which is the amplitude of the imposed rotation, and decreases gradually, following a time course similar to that observed in the rotation alone condition (Fig. 3A, ROT). Values far outside the range of most errors still occur (trials 26, 31, 34, and 65), which indicates that target selection errors continued to occur in the dual task condition. In order to properly identify target errors in the OB+ROT condition we had to take into account the time-varying course of the directional error during rotation learning. We did so by first calculating the average learning curve (*θ* vs. movement number for the ROT condition) for the group of control subjects who learned rotation alone (Fig. 4, white squares). We then took advantage of the fact that directional error in rotation learning follows a decreasing curve that can be accurately fit by a decaying double exponential [26]. We thus fit a double exponential function, *f(x)*, to this average learning curve, where 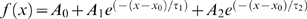; *x* =  movement number; *x_0_* =  movement number at which rotation is first applied. Finally, we defined the plausible range of directional errors for the OB+ROT condition as the set of values from *f(x)−*30° to *f(x)*+30° (shaded region in condition OB+ROT, Fig. 3B). As in the OB condition, errors outside this band were considered target errors.

This algorithm allowed us to identify target errors in the condition that combined rotation and one-back rule (OB+ROT). In order to identify rotation errors in this condition, we computed an *adjusted directional error*, *φ* (Fig. 3C–D). This was defined by calculating directional error relative to each of the three targets, and then selecting the one with the smallest absolute value, if this value was within the plausible range. The angle *φ* is thus the angle between the cursor's path and the direction of the inferred target chosen by the subject (white triangles in Fig. 3B). For trials with correct target selection, *φ* was equal to *θ*. For OB+ROT trials with errors outside the plausible range, *φ* indicated rotation error. (If directional error was outside the plausible range regardless of target selected, then *φ* was undefined).

An example of the calculation of *φ* for a trial in the OB+ROT condition is shown in Fig. 3D. In this trial (trial 31 in Fig. 3B) the correct target was I. The angle (*θ*) between this target's direction and the cursor's path was 56° based on this target. This value for *θ* is outside the plausible range (thick dashed arrow in Fig. 3B). If target II is considered, however, the error is 11° (Fig. 3D), and this is the value assigned to *φ*. This value is not only within the plausible range, but is also similar to neighboring values of directional error along the learning curve. This supports the hypothesis that the subject mistakenly aimed his movement at target II instead of I. The above procedures allowed us to identify, in each condition, errors attributable to implicit (rotation errors) and explicit control processes (target errors).

## Results

### Normal Dual-Control Task Performance in CTL Group

Control subjects performed the dual-control task without interference. The time course of directional error for individual subjects in each of the four conditions tested in our study is shown in Fig. 3 (described in detail in *Materials and Methods*). Group data is shown in Fig. 4 for subjects in the ROT condition vs. OB+ROT. Directional error gradually decreased from as subjects adapted to the imposed rotation (Fig. 4A; 1 cycle = 3 trials). The course of adaptation was indistinguishable between the two groups. We confirmed this statistically by comparing, between the OB and OB+ROT groups, the average values of directional error for the first 6 cycles (*p* = 0.54; 2-sample *t* test), as well as for the last 4 cycles (*p* = 0.37) of rotation learning. Thus concurrent performance of the one-back task with rotation learning did not interfere with rotation learning. Note that, although we did not measure after-effects in this protocol, we limited our measure of learning to learning rate and total amount of learning. In this study, lack of interference thus refers to lack of degradation of learning performance between two tasks.

There was also no detectable effect of rotation learning on the one-back task. Error rates for target selection were 1.4%±0.8 (mean±SE) in the OB condition, and 1.9%±0.8 in the OB+ROT condition (Fig. 4B), a difference that was not statistically significant (*p* = 0.21; paired *t* test). These results show that reaching direction can be influenced by parallel implicit and explicit control processes, with successful segregation of overlapping sensorimotor signals.

### Normal Movement Execution in AC Group

AC subjects made straight movements with bell-shaped velocity profiles, similar to those of control subjects. There was no significant difference between groups, across all conditions, in movement kinematics, including measures of within-movement corrections and movement preparation time (*p*>0.05; ANOVA with group, condition, and their interaction as factors; see Supporting Text S1).

### Normal Single-Task Performance in AC Group

**Figure 5:**
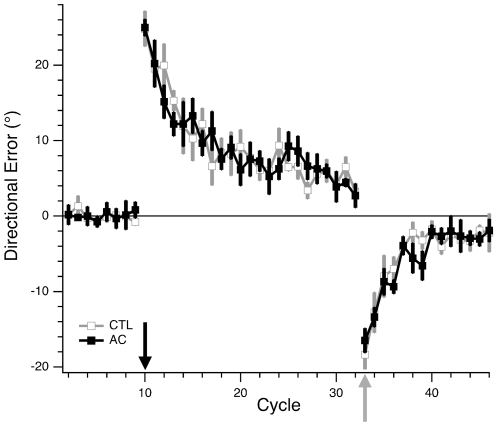
Learning curves for rotation learning in the dual-control conditions for CTL and AC subjects. Both traces show the directional error (*θ*), averaged within each cycle (1 cycle = 3 trials) and across all subjects in the respective groups, vs. cycle number. Open squares, grey trace, CTL (group I); filled squares, black trace, AC (group II). The first 9 cycles were in the BL condition. For the next 23 cycles a 30° CCW rotation was imposed (black arrow; ROT condition). The remaining trials were in the BL condition (grey arrow; de-adaptation period).

AC subjects performed normally in single-control tasks. In the ROT condition, AC subjects gradually reduced their directional error similarly to control subjects: their adaptation curves overlapped throughout the learning period (Fig. 5, cycles 10–32). There was no significant difference, between the CTL and AC groups, in the average values of directional error for the first 6 cycles (*p* = 0.79; 2-sample *t* test) of rotation learning, as well as for the last 3 cycles (*p* = 0.32).

Rotation learning was followed by a de-adaptation period, in which the BL condition was reintroduced, for 42 trials (Fig. 5, cycles 33–46). Removal of the imposed rotation produced an “after-effect”, i.e. directional error in the opposite direction to the previously imposed rotation, as previously described [27]. The after-effect curve for AC subjects closely followed that of controls. Average directional error was the same for AC and CTL groups in the first cycle of the de-adaptation period (*p* = 0.56; 2-sample *t* test), as well as in cycles 2–6 of this period (*p* = 0.42). Note that the gradual decay of the after-effect is evidence of the implicit nature of rotation learning in AC and control subjects. If subjects had used an explicit strategy to counter the effects of rotation, the after-effect should not have persisted for more than a few trials, followed by a switch back to the baseline strategy.

The AC group also performed similarly to controls in the OB condition, making target selection errors on only 3.4% of the trials (mean±SD: 3.4%±1.8), which was not significantly different from control subjects' error rate (1.4%±0.8; 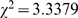, p = 0.114).

### Impaired Dual-Task Performance in AC Group

**Figure 6:**
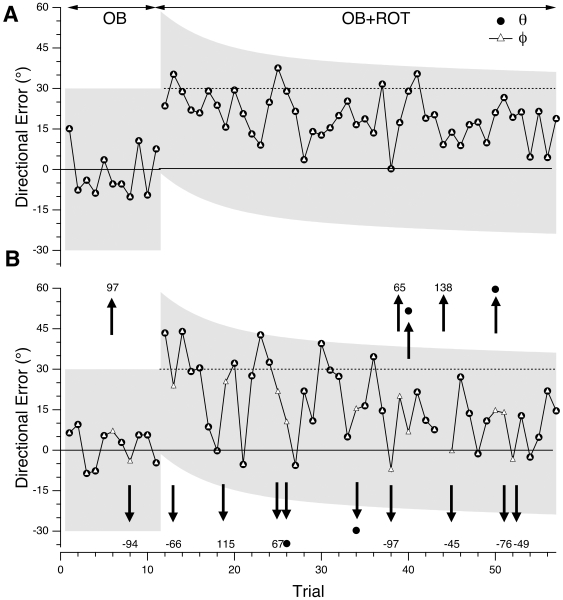
Directional error of two representative AC subjects in a series of trials in the single and dual one-back reaching task. Directional error (*θ*) and adjusted directional error (*φ* ), defined as in Fig. 3, are plotted against movement number for a portion of a testing session. Directional error, *θ*, and adjusted directional error, *φ*, are plotted against movement number for individual subjects. Trials 1–10 were in the OB condition (no rotation); the remaining trials were performed with a 30° CCW rotation (OB+ROT condition). (A) Data for an AC subject (group IV) who made no target selection errors (*φ* = *θ* for all trials). (B) Data for an AC subject (group IV) who made several target selection errors (arrow). Numbers near the arrows indicate value of *θ* for those trials.

When AC subjects had to learn rotation while simultaneously following the one-back instruction (OB+ROT condition), their performance deteriorated. The extent of deterioration in rotation learning and one-back performance varied from subject to subject. Fig. 6 shows single-subject examples of OB+ROT performance abnormalities. The first subject (Fig. 6A) made no target errors in either OB or OB+ROT conditions. When the 30° rotation was imposed (OB+ROT condition; trial 12), directional error increased to values near 30°. It then gradually decreased, but comparison with Fig. 5 indicates that this learning proceeded more slowly compared to when AC subjects learned rotation without the one-back component of the task. In the latter case, the group's directional error decreased below 10° after 24 trials (i.e., after 8 cycles of learning; see cycle 18 in Fig. 5) and remained steadily below this value as training continues. Directional error for the subject in Fig. 6A, on the other hand, remained above 10° (on average) even after 40 trials. This subject's performance is also in contrast with that of control subjects performing the dual-control task. Errors for the single subject in Fig. 3B, for example, decreased below 10° after 21 trials, and stayed below this value for most of the remainder of the learning period.

Fig. 6B illustrates, for another AC subject, performance deterioration that was mostly in target selection. As in Fig. 2, the arrows mark trials where target selection errors occurred. These are instances where the directional error, *θ*, was outside the range of plausible errors (shaded area in Fig. 6). For most error trials, the plot shows a value for adjusted directional error (*φ*). The exception is trial 44, where *φ* is not defined because the directional error (138°) cannot be reduced to the plausible range by replacing the trial's target with either of the other two targets (see Methods). In contrast to the subject in Fig. 6A, this subject made target selection errors more frequently: two occurred in the 9 movements of OB condition shown, and 13 occurred among the 46 OB+ROT trials shown. Error rates across all trials in each epoch for this subject were 18% for the OB condition, and 35% for the OB+ROT condition.

**Figure 7:**
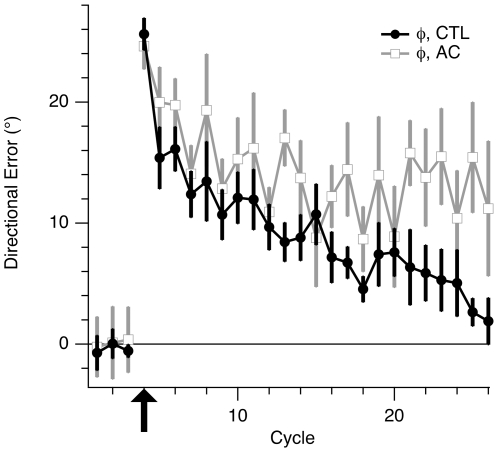
Learning curves of CTL and AC subjects learning rotation in the dual-control conditions. Filled circles (black trace) show the adjusted directional error (*φ* ), averaged within each cycle (1 cycle = 3 trials) and across all subjects in group III (CTL), vs. cycle number in the OB+ROT condition. This is the same trace as shown in Fig. 4 (filled circles in that figure). Open squares (grey trace): same information, but for subjects in group IV (AC). The first three cycles were performed without rotation. On cycle 4 a 30° CCW rotation was imposed for the remainder of the session (black arrow).

Fig. 7 shows the average learning curves for the OB+ROT conditions for AC and CTRL subjects. Directional error for control subjects (same as black trace with filled circles in Fig. 4) decreased steadily throughout the learning period. Errors of AC subjects, on the other hand, initially decreased, and then stabilized at values well above those of the CTRL group. The large error bars indicate great variation in performance across subjects. One-back performance also worsened during ROT in AC subjects, increasing from 3.4%±1.8 in the OB condition to 23%±11 in the OB+ROT condition (Fig. 4B; *p* = 0.004).

**Figure 8:**
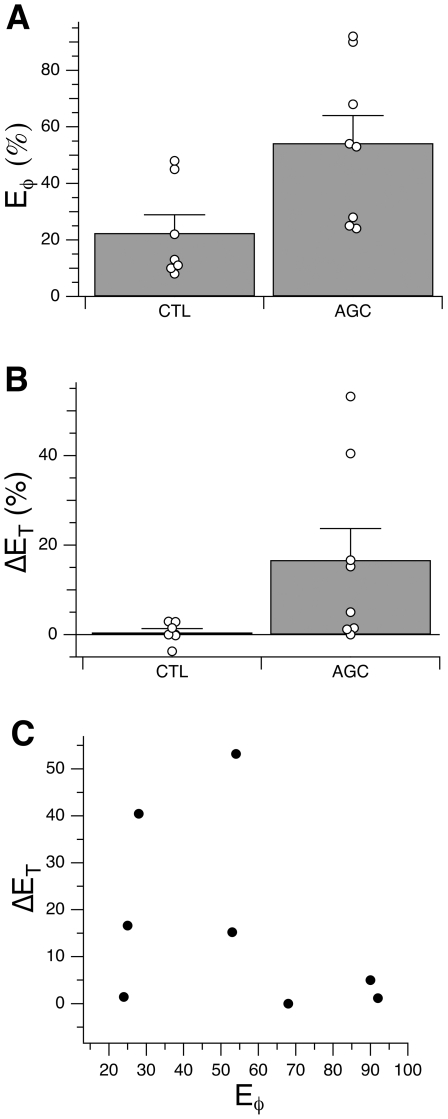
Performance of CTL and AC subjects on the dual task. (A) Percent remaining directional error (*Eφ*) in the last 3 cycles of rotation learning in the OB+ROT condition (100% = 30°). (B) Change in frequency of target selection error (Δ*E_T_*) from the OB to the OB+ROT conditions. This is expressed as a difference between the percentage of errors in each condition. For (A) and (B), subject groups are III (CTL) and IV (AC); circles indicate values for individual subjects; bars show group mean±SE. (C) Relationship between target selection error and rotation error in the dual-control task for AC subjects. The plot shows the change in frequency of target selection error (Δ*E_T_*) from the OB to the OB+ROT conditions vs. percent remaining directional error (*Eφ*) in the last 3 cycles of rotation learning in the OB+ROT condition.

We calculated single measures to summarize the amount of rotation learning and one-back performance. For rotation learning, we chose the remaining directional error at the end of the learning period. For each subject, we calculated the average adjusted directional error (*φ*) from trial 52 through 60, i.e. the directional error averaged over cycles 17 through 19. We then divided this value by the imposed rotation (30°), and obtained *Eφ* , the residual directional error as a fraction of the total amount of learning required, which could range from 0 (indicating 100% adaptation) to 100% (indicating no adaptation). For one-back performance, we computed the change (Δ*E_T_*) in the frequency of target selection errors (*E_T_*) from the OB condition to the OB+ROT condition (Δ*E_T_  =  E_T(OB+ROT)_ − E_T(OB)_*). These measures are shown in Fig. 8.

Residual directional error (*Eφ* ) was significantly larger in the AC group (54%±10; mean±SE) than in the CTL group (22%±6; *p* = 0.02, 2-sample *t* test; Fig. 8A). The change in frequency of target selection error (*ΔE_T_*) was 0.5%±0.9 for controls (mean±SE) and 17%±7 for the AC group (Fig. 8B). We tested for the effect of group and condition on *E_T_* in an ANOVA design with group (CTL vs. AC) as a between-subject measure and condition (OB vs. OB+ROT) as a repeated measure. We first applied a square-root transformation to the data in order to obtain normally distributed samples. There was a significant effect of condition (*p* = 0.001), no overall effect of group (*p* = 0.11), and a significant group by condition interaction (*p* = 0.02). Post-hoc paired *t* tests revealed that *E_T_* did not change significantly between the OB and OB+ROT conditions for the CTL group (*p* = 0.21), while it increased significantly for the AC group (*p* = 0.004). In other words, as indicated by the significant interaction term and confirmed by post-hoc testing, the frequency of target selection errors increased significantly in the dual version of the task only for the AC group.

### Role of Cognitive/Attentional Factors

We considered the possibility that the AC subjects' impairment in the dual-control task might be due to an impairment of attention, rather than to a failure to separate implicit and explicit control processes. One argument against this possibility is the fact that, as we previously demonstrated, attentional processes do not seem to contribute to rotation learning [10]: this type of adaptation proceeds unaffected even when conscious effort is made to prevent it from happening. The dual-control task, therefore, is designed not to tax attention any more than the individual tasks do. Given that AC subjects can perform the individual tasks correctly, it is difficult to envision how impaired attention could disrupt a dual-control task that does not require more attention than the single tasks.

We also looked for evidence of a contribution of a non-specific attentional or cognitive deficit to the AC group's dual-task impairment. Such a deficit would be expected to affect overall performance of the task by a certain amount, but should not have specific effects on the separate components of a dual task. A given total attentional impairment would be expected to affect either mostly one task, or mostly the other, or each one of them partially. Across multiple subjects, therefore, there should be a negative correlation between deficits in one task and the other. If we assume that all AC subjects have a similar overall attention deficit, then a negative correlation would be expected between rotation and target errors: subjects whose performance in one task is affected more than average would be expected to be affected less than average on the other task. This possibility was not supported in the present study: there was no significant (positive or negative) correlation between target errors (Δ*E_T_*) and rotation error (*Eφ*) (*R^2^* = 0.13, *p* = 0.38; Fig. 8C). Our sample size (N = 8 subjects) yielded 80% power to detect *R^2^* as low as 0.36. Of course, this prediction would not be not valid if there were sufficient inter-subject variability in severity of the hypothesized cognitive/attentional deficit. Therefore, while a negative correlation would be in support of an attentional deficit, lack of negative correlation does not entirely exclude this possibility.

The observed deficits are unlikely to be due to sensorimotor difficulties, such as trajectory control deficits or misperception of target direction. AC subjects had no movement abnormalities on clinical examination. Moreover, their movement kinematics were normal in all conditions (Supporting Text S1).

It is also unlikely that the source of impairment lies in inadequate time for sensory processing and motor planning. Although subjects were instructed to make fast movements, they were also instructed to start their movements only when they felt ready to do so. Thus the task was not a reaction-time paradigm. Indeed, mean movement preparation time ranged from 660 to 900 ms across conditions (Supporting Text S1). These intervals are much longer than typical reaction times in simple or choice tasks (200–350 ms), which argues against a limitation on processing time. More importantly, movement preparation times were not different between subject groups (Supporting Text S1).

### Relationship to Pre-clinical State

We tested for correlation between AC subjects' dual-control impairment and estimates of how close subjects were to time of disease onset. We considered the estimated number of years before diagnosis, *T_d_* [30], [31], [32], and the probability of diagnosis in five years' time, *P_5y_* [30]. Our two groups of AC subjects (groups II and IV) did not show significant differences in these measures (*T_d_* mean±SD 15±10 years for group II, 20±9 years for group IV; *P_5y_* 0.17±0.16 for group II, 0.09±0.10 for group IV). There was no significant correlation between each performance variable in the double-control task (residual directional error, *Eφ*; change in rate of target selection error, Δ*E_T_*) and either *P_5y_* or *T_d_* (*p*>0.1).

This result must be interpreted with caution. First, the clinical diagnosis of HD is entirely based on motor manifestations, and thus estimates of time-to-diagnosis do not necessarily reflect the time when cognitive and psychiatric symptoms appear. Given that the striatum participates in multiple neural processes and that the manifestations and time course of clinical symptoms of HD exhibit great inter-individual variation, it is not clear whether the disease's effect on one aspect of behavior should correlate with the probability of clinical diagnosis. Second, estimates of time-to-diagnosis are accompanied by large variance [30], which limits their applicability to individual subjects' data, especially given the small numbers of subjects in our groups.

## Discussion

The present study addressed the human motor system's ability to control the same action implicitly or explicitly. Using a dual-control reaching task in which implicit and explicit control could be separately monitored, we tested the independence of these two control processes. Healthy control subjects were able to exert dual explicit and implicit control without interference, while subjects with the genetic mutation for a neurologic disorder that causes degeneration of the striatum and related circuits, but without clinical abnormalities, were impaired in dual control of action.

These results demonstrate that explicit and implicit processes can share control of reaching without mutual interference. This would not be surprising for dual tasks with separate responses (Fig. 1A), such as those employed in studies of learning and memory [3], [4]. In a dual-control, single-action task, however, the ability to maintain parallel control is remarkable, because overlapping sensorimotor information must guide a single behavior. The distinction between implicit and explicit processes, originally formulated for learning and memory, can thus be extended to the domain of action guidance and motor control, as has been suggested [2], [5], [33]: just as explicit and implicit processes can parse a single experience into separate declarative memory and procedural learning components [3], so can such processes separately influence a single action.

The need to segregate sensorimotor signals is supported by the fact that brain networks supporting the tasks used in this study are distinct. Visuomotor adaptation requires computations involving posterior parietal cortex, premotor cortex, pre-supplementary motor area, and cerebellum, as well as basal ganglia [34], [35], [36], while working memory processes underlying one-back reaching are associated with activation of premotor, supplementary motor, prefrontal, cingulate, posterior parietal cortex, and caudate [33], [37], [38], [39]. If these separate neural processes are to operate on the same spatial information and guide the limb, then sensory information must diverge to different control processes, and motor information from multiple processes must be merged. This divergence-convergence of sensorimotor signals could take place in a structure with the striatum's connectivity. Striatal degeneration could thus result in cross-talk among signals from different brain regions, or incorrect sorting of sensorimotor signals to different control processes, and lead to impaired performance when explicit and implicit control are concurrently engaged.

Our results suggest that segregation of explicit and implicit motor control is a distinct capacity of neural systems that underlie sensorimotor behavior, dissociable from the control processes themselves. The fact that this deficit was found in the presymptomatic stages of HD suggests the striatum as a possible neural substrate for segregation of motor control processes. In patients with manifest HD, the degree of severity of clinical signs is correlated with imaging measures of striatal dysfunction [40]. There is also ample evidence of subclinical structural and functional changes in the basal ganglia in the presymptomatic stage [41], [42], [43], [44], [45]. Although neuropsychologic abnormalities have been reported in AC individuals (e.g., [46], [47]), there is no firm evidence of such abnormalities when the asymptomatic state is confirmed through a neurologic evaluation [48], [49], [50], [51]. Our strict selection of asymptomatic subjects with normal neurologic examination thus allowed us to test the ability to segregate explicit from implicit motor control in a group of subjects with incipient degeneration of the striatum but without sensory, motor, or cognitive impairments.

Diseases that disrupt processing in the striatum, such as HD and Parkinson's disease (PD), are known to impair control of multiple tasks (reviewed in [52]), and functional imaging studies in healthy subjects have implicated the striatum in dual-task performance [53]. Our results offer a possible specific abnormality (disrupted segregation of signals to multiple control systems) as the underlying pathophysiology of multi-tasking in these diseases. A role of the striatum in the segregation of explicit and implicit control is consistent with the striatum's proposed “filtering” role in selecting desired movements and suppressing similar but unwanted ones [54], because proper routing of sensorimotor signals may be required for such selection. Disruption of dual-control segregation could result from damage to striatal neurons receiving cortical inputs (medium spiny neurons), which indeed degenerate early in HD [11]. The impairment observed in AC subjects is thus consistent with a role of the striatum in sensorimotor signal segregation, given this structure's connectivity and known dysfunction/degeneration in the presymptomatic stage [41], [42], [43], [44], [45]. However, we cannot exclude the possibility that this deficit may also be due to HD's effects on other brain regions [12].

The striatum is known to play other roles in motor control. Abnormalities in the trajectories of reaching movements and response to movement perturbations, observed in patients with HD, suggest a role in guidance and corrections of ongoing movements [15]. The AC subjects' normal adaptation to a visuomotor rotation in our study is consistent with the previously described intact ability to learn a new internal model [55], and suggests that the striatum does not play a crucial role in trial-by-trial adaptation. Symptoms such as bradykinesia and hypokinesia seen in PD suggest a role in the selection of movement amplitude and speed based on energy requirements [56]. A role for the striatum in segregation of sensorimotor signals may be unrelated to these functions, but may explain some of the difficulties with “multi-tasking” that have been reported with HD [17], [57] and PD [58], [59], [60].

The impairments we identified in AC subjects may be viewed as a deficit in dual-task control. This type of control likely involves other processes besides sensorimotor signal segregation, and it is thus possible that the underlying deficit reflects other aspects of dual task control. It is unlikely that perceptual and execution deficits can explain the observed dual-control deficits, given normal performance of single-control versions of the task components. The fact that visuomotor rotation is immune to attentive control [10] makes it unlikely that the observed deficits were due to general difficulties in dividing attention. However, while a deficit in sensorimotor signal segregation offers a consistent explanation of our results, a non-specific difficulty with dual control, unrelated to sensorimotor signal segregation, cannot be entirely excluded as a possible explanation.

Although the dual-control task did not impose specific constraints on timing of the two control processes, the lack of interference is unlikely to be explained by sequential engagement of explicit and implicit control. The one-back task required remembering target direction for the previous target and the current target from one trial to the next, and updating these when the next target appears and after the current movement has been planned. Thus the explicit control process needed to be active (at least by maintaining proper labeling of its relevant sensorimotor signals) throughout the interval from one movement to the next. Rotation learning required comparison of cursor direction to target direction, followed by an adjustment to the sensorimotor map. This adjustment likely occurs in the immediate period after each movement [61], which implies that the dual-control task required a comparison of directions to be performed while explicit control maintained its own set of directions in working memory. Thus the relevant sensorimotor signals needed to be maintained simultaneously, and interference would have resulted without a system for segregating direction signals to implicit and explicit control processes. However, an alternative possibility, namely, that AC subjects' difficulty with the task stemmed to some extent from the sequential handling of sensorimotor information, cannot be fully excluded.

The present study demonstrates that implicit and explicit motor control can guide movements independently, without interference. This ability may allow the motor system to vary the amount of explicit and implicit motor control based on task requirements. A possible role for this flexibility (Willingham's “dual mode” principle) [5], [6] has been postulated in motor skill learning, in which explicit control may be engaged to modify automatically controlled movements.

## Supporting Information

Text S1 Analysis of Kinematic Variables
(0.09 MB DOC)
