# Supplementary material for: Parallel Explicit and Implicit Control of Reaching
Source: PLoS One. 2009 Oct 22;4(10):e7557. doi: 10.1371/journal.pone.0007557 (PMC2760763; doi:10.1371/journal.pone.0007557)
Supplement: Text S1 — Analysis of Kinematic Variables (0.09 MB DOC) [file pone.0007557.s001.doc]

We calculated additional kinematic measures as follows. *Movement duration*: interval between movement start and end times. *Peak velocity*: first maximum in the velocity profile above 15 cm/s. *Time to peak velocity*: interval from movement start to peak velocity. *Velocity profile asymmetry*: contrast of deceleration vs. acceleration time, calculated as their difference (deceleration - acceleration time) divided by their sum (deceleration+ acceleration time). Deceleration is defined as the time elapsed from peak velocity to movement end, and acceleration as the time from movement start to peak velocity. This quantity ranges from -1 to 1 and is affected by corrections along movement direction as the hand nears the target. *Path curvature*: total unsigned area between the path followed by the hand (from movement start to end) and a straight line connecting the hand's start and end positions; this area is then normalized by dividing it by the area of a semicircle of diameter equal to the distance between movement start and end points. This is a sensitive measure for any deviations of the handpath from linearity, including corrective changes in direction. *Direction change*: difference between movement direction at movement end and movement direction at peak velocity. This is a measure of directional corrections in the second portion of the movement. *Movement preparation time*: interval between target appearance and movement start. Note that this is not equivalent to reaction time, as the instruction was not to start moving as soon as possible, but rather to move when ready to do so.

There was no significant difference between groups in movement kinematics in all conditions. Kinematic measures included movement duration, peak velocity, time to peak velocity, extent error, directional error, velocity profile asymmetry, path curvature, direction change within movement, and movement preparation time (p < 0.05; ANOVA with group, condition, and their interaction as factors; Supporting Table S1). There were significant effects of condition (Supporting Table S1, below) on: directional error (as expected, given the externally induced error in ROT, OB+ROT); path curvature and direction change (indicating greater amount of within-movement corrections in ROT, OB+ROT); movement preparation time (shorter times in OB, OB+ROT). However, even for these variables, there was no interaction effect between group and condition.

| **Supporting Table S1. Kinematic variables across groups and conditions** | | | | |  | |  | |
| --- | --- | --- | --- | --- | --- | --- | --- | --- |
| **Variable** | **Condition** | **Group** | | **ANOVA** | | | | |
|  |  | **CTL** | **AC** | Factor | | F value | | p value |
| Movement Duration | BL | 530 ± 161 | 700 ± 121 | Condition | | 1.72 | | N.S. |
| *(ms)* | ROT | 542 ± 161 | 563 ± 131 | Group | | 1.66 | | N.S. |
|  | OB | 673 ± 121 | 711 ± 144 | Cond. X Group | | 0.77 | | N.S. |
|  | OB+ROT | 646 ± 121 | 642 ± 131 |  | |  | |  |
| Peak velocity | BL | 26 ± 8 | 22 ± 6 | Condition | | 0.45 | | N.S. |
| *(cm/s)* | ROT | 28 ± 8 | 26 ± 7 | Group | | 0.49 | | N.S. |
|  | OB | 28 ± 6 | 25 ± 7 | Cond. X Group | | 0.22 | | N.S. |
|  | OB+ROT | 27 ± 6 | 28 ± 7 |  | |  | |  |
| Time to Peak Velocity | BL | 255 ± 89 | 324 ± 67 | Condition | | 1.55 | | N.S. |
| *(ms)* | ROT | 275 ± 89 | 270 ± 73 | Group | | 2.19 | | N.S. |
|  | OB | 313 ± 67 | 370 ± 80 | Cond. X Group | | 0.47 | | N.S. |
|  | OB+ROT | 308 ± 67 | 328 ± 73 |  | |  | |  |
| Extent Error | BL | -0.2 ± 1.1 | 0.2 ± 0.9 | Condition | | 0.88 | | N.S. |
| *(cm)* | ROT | 0.4 ± 1.1 | 0.3 ± 0.9 | Group | | 0.02 | | N.S. |
|  | OB | 0.6 ± 0.9 | 0.9 ± 1 | Cond. X Group | | 0.51 | | N.S. |
|  | OB+ROT | 0.8 ± 0.9 | 0.3 ± 0.9 |  | |  | |  |
| Directional Error | BL | 0 ± 4 | -1 ± 3 | Condition | | 34.46 | | < 0.001 |
| *(degrees)* | ROT | 9 ± 4 | 7 ± 3 | Group | | 0.36 | | N.S. |
|  | OB | -1 ± 3 | 0 ± 4 | Cond. X Group | | 2.14 | | N.S. |
|  | OB+ROT | 10 ± 3 | 15 ± 3 |  | |  | |  |
| Velocity Profile Asymmetry | BL | 35 ± 138 | 39 ± 104 | Condition | | 0.83 | | N.S. |
|  | ROT | -57 ± 138 | 19 ± 112 | Group | | 0.26 | | N.S. |
|  | OB | 43 ± 104 | 38 ± 123 | Cond. X Group | | 0.24 | | N.S. |
|  | OB+ROT | -19 ± 104 | -20 ± 112 |  | |  | |  |
| Path Curvature | BL | 0.28 ± 0.1 | 0.32 ± 0.07 | Condition | | 5.43 | | < 0.001 |
|  | ROT | 0.31 ± 0.1 | 0.35 ± 0.08 | Group | | 0.98 | | N.S. |
|  | OB | 0.19 ± 0.07 | 0.19 ± 0.09 | Cond. X Group | | 0.18 | | N.S. |
|  | OB+ROT | 0.23 ± 0.07 | 0.26 ± 0.08 |  | |  | |  |
| Direction Change | BL | -1 ± 2 | 1 ± 1 | Condition | | 6.54 | | < 0.001 |
| *(degrees)* | ROT | -2 ± 2 | -3 ± 1 | Group | | 0.39 | | N.S. |
|  | OB | 0 ± 1 | 0 ± 1 | Cond. X Group | | 2.42 | | N.S. |
|  | OB+ROT | -2 ± 1 | -2 ± 1 |  | |  | |  |
| Movement Preparation Time | BL | 750 ± 207 | 897 ± 157 | Condition | | 2.73 | | N.S. |
| *(ms)* | ROT | 784 ± 207 | 891 ± 169 | Group | | 0.91 | | N.S. |
|  | OB | 707 ± 157 | 670 ± 185 | Cond. X Group | | 0.61 | | N.S. |
|  | OB+ROT | 658 ± 157 | 656 ± 169 |  | |  | |  |
| CTL, control subjects. AC, asymptomatic carriers. All kinematic variables listed as mean ± SD | | | | | | | |  |
| BL, baseline; ROT, rotation; OB, one-back | | | | |  | | |  |
| N.S., not significant. See Methods and Supplementary Text S1 for definitions | | | | |  | | |  |
